# New Work Forthcoming

**Published:** 1857-02

**Authors:** 


*New Work Forthcoming.*—We are gratified to learn that Prof. Austin Flint is engaged in the preparation of a work on the "Diagnosis, Pathology and Treatment of Diseases of the Heart." We are not informed how soon it may be expected, but are confident that it will be eagerly welcomed by the profession. Messrs. Blanchard & Lea, of Philadelphia, are to be the publishers.

